# Concurrent chemoradiation with capecitabine and weekly irinotecan as preoperative treatment for rectal cancer: results from a phase I/II study

**DOI:** 10.1038/sj.bjc.6603053

**Published:** 2006-03-21

**Authors:** G Klautke, U Küchenmeister, T Foitzik, K Ludwig, F Prall, E Klar, R Fietkau

**Affiliations:** 1 Department of Radiotherapy, University of Rostock, University Hospital, Südring 75, 18059 Rostock, Germany; 2 Department of Surgery, University Hospital, Rostock, Germany; 3 Department of Surgery, Klinikum Südstadt, Rostock, Germany; 4 Department of Pathology, University Hospital, Rostock, Germany

**Keywords:** rectal cancer, neoadjuvant chemoradiotherapy, capecitabine, irinotecan

## Abstract

The aim of this study was to investigate the efficacy and safety of chemoradiation using capecitabine and irinotecan as neoadjuvant therapy for patients with rectal cancer. Conventional radiation was given at daily fractions of 1.8 Gy on 5 days a week for a total dose of 55.8 (50.4+5.4) Gy. Concurrently, irinotecan 40 mg m^−2^ once weekly and capecitabine continuously at dose levels of 500, 650, 750 and 825 mg m^−2^ twice daily were administered. Surgery was performed 4–6 weeks following completion of chemoradiation. A total of 28 patients (3 UICC II, 25 UICC III) were enrolled and all received treatment. Dose-limiting toxicity was diarrhoea grade IV and hand–foot syndrome at the 825 mg m^−2^ dose level. The maximum tolerated dose of capecitabine was 750 mg m^−2^. Diarrhoea was the most common toxicity: grade III in nine patients. Two patients died, one due to pneumonia and one due to sudden cardiac death. A complete response and only microfocal residual tumour disease was achieved in four and three patients (27%). In all, 25 of 28 patients undergoing surgery, 24 (96%) had R0 resection. Preoperative chemoradiation based on continuous daily capecitabine and weekly irinotecan appears to tolerated and effective in patients with rectal cancer.

Achieving tumour resection with clear margins (R0) is the most important prognostic factor in rectal carcinoma and the principal objective of treatment (Hermanek and Wittekind, 1994) while maintaining faecal continence is an important objective for maintaining patients’ quality of life. In locally advanced rectal cancer (LARC), it can be difficult or impossible to achieve both of these objectives and local recurrence and metastases can be a major problem following curative surgery (Hurby *et al*, 2003). Radiotherapy or chemoradiation has been widely used to improve patient outcomes in locally advanced rectal cancer (LARC), 5-FU-based chemoradiation is an effective treatment, shown by many phase II studies (Chen *et al*, 1994; Kaminsky-Forrett *et al*, 1998; Janjan *et al*, 1999; Crane *et al*, 2003). Preoperative chemoradiation increases the resection rate (Reerink *et al*, 2003) and preoperative radiation or chemoradiotherapy decrease local recurrence and reduce small bowel complications compared with postoperative therapy (Minsky *et al*, 1992; Frykholm *et al*, 1993, Sauer *et al*, 2004). R0 resection rates of 60–85% have reported with 5-FU-based preoperative chemoradiation for LARC (Videtic *et al*, 1998; Küchenmeister *et al*, 2000; Rödel *et al*, 2000).

Continuous infused application of 5-FU significantly improves overall and disease-free survival compared with bolus administration of 5-FU (O’Connell *et al*, 1994). However, infused application is inconvenient and is associated with an increased risk of adverse venous events, such as infection or thrombosis, and most cancer patients prefer oral chemotherapy (Liu *et al*, 1997). Capecitabine, an oral, tumour-activated fluoropyrimidine carbamate, delivers 5-FU preferentially to tumour cells via enzymatic conversion. The final step of this process is mediated by the enzyme thymidine phosphorylase (TP), which is significantly upregulated in tumour tissue compared with healthy tissue (Schüller *et al*, 2000). TP expression is also enhanced by radiotherapy and the *in vivo* antitumour activity of radiotherapy and capecitabine is more than additive compared with either agent alone (Sawada *et al*, 1999). Twice-daily oral administration of capecitabine enables chronic dosing that results in continuous exposure to 5-FU without requiring central venous access. As first-line therapy for MCRC, capecitabine results in superior response rates, improved safety, and improved convenience compared with 5-FU/LV (Mayo Clinic regimen) (Van Cutsem *et al*, 2004). Capecitabine was also continuously applicated, concurrent to radiotherapy preoperatively with good results (Dunst *et al*, 2002).

The topoisomerase I inhibitor irinotecan has shown consistent efficacy in both chemotherapy-naive patients and 5-FU-pretreated patients with metastatic colorectal cancer (Douillard *et al*, 2000; Saltz *et al*, 2000). Also radiosensitizing properties even under hypoxic conditions were documented (Boothmann *et al*, 1987; Boscia *et al*, 1993), and Irinotecan has also shown in combination with 5-FU and radiation preoperatively in patients with LARC consistent efficacy in local control and DFS (Mehta *et al*, 2003; Klautke *et al*, 2005). As a result of that, it was a logical step to investigate the combination of capecitabine and irinotecan in patients with LARC.

The aim of this study was to define the maximum tolerated dose (MTD), dose-limiting toxicity (DLT) and safety profile of capecitabine in combination with irinotecan and radiotherapy as preoperative chemoradiation in patients with LARC.

## PATIENTS AND METHODS

This was an open-label, 6-week study of increasing dose levels of capecitabine given concurrently with irinotecan and standard whole pelvic irradiation as preoperative therapy for rectal cancer clinical UICC stage II or III.

### Patient eligibility

Male and female patients aged 18–75 years were prospectively enrolled in the study if they presented with histologically confirmed nonmetastatic adenocarcinoma of the rectum at an UICC stage II or III. Other inclusion criteria were measurable disease (at least one bidimensionally measurable tumour lesion), WHO performance status ⩽2, adequate haematological, hepatic and renal function, and a life expectancy of at least 3 months. Pregnant or lactating women, patients with unresolved bowel obstruction or ileus/subileus and those with a history of chronic diarrhoea were excluded.

All patients underwent baseline examination and staging according to the recommendations of the German Cancer Society within 2–4 weeks prior to the start of chemoradiation, including history and physical examination, complete blood count, serum chemistry profile, chest X-ray, rectoscopy or sigmoidoscopy, endoluminal ultrasound, abdominal ultrasound and computed tomography (CT) of the abdomen and pelvis. Inclusion of a minimum of 10 patients in the phase II part and a minimum of 15 patients at the recommended dose level was planned.

The study was approved by the local ethics committee, and written informed consent was obtained from all patients.

### Treatment

CT-assisted 3-dimensional planning of radiation therapy was employed. The patients underwent CT with 5 mm slices, contrast administration to bladder, rectum and small intestine, and endoscopic clipping (Riepl *et al*, 2000) of the upper and lower border of the tumour performed immediately prior to planning. Radiation therapy was given with photons from a linear accelerator with energy >6 MV. The target volume comprised the areas at risk including the presacral space along the posterior bladder or vaginal wall, respectively, and the common iliac lymph nodes until and including the fifth lumbar vertebral body. Radiotherapy was delivered with three or four fields using an isocentric technique with individually collimated field portals. Daily fractions of 1.8 Gy (calculated at the ICRU 50 reference point) were given on 5 days a week over 5 weeks to a total dose of 50.4 Gy. An additional low-volume boost of 5.4 Gy was given in three fractions to the site of the primary tumour after previous contrast radiography of the small intestine.

Radiotherapy was administered with concurrent chemotherapy consisting of weekly doses of irinotecan 40 mg m^−2^, given over 90 min on days 1, 8, 15, 22, 29 and 36. Patients also received capecitabine twice daily at 12-h intervals at the following dose levels: 500, 650 and 825 mg m^−2^. As the increase from the 650 to the 825 mg m^−2^ twice daily capecitabine dose level proved to be too large during the course of the study, in an amendment a further dose level of 750 mg m^−2^ twice daily was introduced before the dose was increased to 825 mg m^−2^. So all steps of increasing the dose from 500 mg m^−2^ to 825 mg m^−2^ twice daily capecitabine were performed adequately.

At the first dose level three patients were planned. If no DLT occurred, again three patients were planned at the next dose level. If any DLT occurred, another three patients were treated at the same dose level. If there was again a DLT, the level below was the recommended dose level. If there was no DLT another three patients at the next dose level were planned.

Patients were monitored by history, clinical examination and blood examination on every Monday and Thursday during the treatment period.

The chemotherapy was interrupted if any DLT occurred and was continued at 75% of the original dose when toxicity resolved to grade I or II. In a case of diarrhoea grade IV radiation and chemotherapy were interrupted and were continued when diarrhea resolved to grade II with radiation and 50% of the original dose of chemotherapy.

Restaging and surgery using total mesorectal excision was to be performed within 4–6 weeks after completion of chemoradiotherapy. Following surgery, a postoperative adjuvant chemotherapy according to the recommendations of the German Cancer Society was recommended to all patients.

### Dose-limiting toxicity

Toxicity was graded according to the National Cancer Institute Common Criteria (NCI-CTC, version 3.0). Defined DLTs included: grade IV diarrhoea despite appropriate treatment with antidiarrhoea drugs; grade IV haematological side effects; grade IV mucositis; any other acute side effect at grade IV; severe nausea; vomiting 6–10 times a day despite the use of antiemetics.

### Data evaluation

The deadline for data evaluation was June 30, 2005. Statistical analysis including survival analysis according to Kaplan–Meier was performed with the SPSS software package. Survival was calculated from the date of histologic verification of diagnosis to the patient's death or the date of last follow-up. Progression-free survival was calculated from diagnosis to the time of first detection of new lesions or progression of residual lesions.

## RESULTS

### Patient characteristics

Between July 2002 and May 2004, we recruited 28 patients (16 women and 12 men) aged from 48 to 75 years (median, 64 years) who met all the inclusion criteria. Baseline patient and tumour characteristics are shown in Table 1. There were two cases of a primary tumour stage uT2, all lymph node positive, 18 cases of uT3, all beside two lymph node positive and eight cases at stage uT4, seven endosonography assessed the lymph node status as positive. The median length of the tumour, as an approximate measure of the tumour mass, was 7 cm (range 3–15 cm). In all, 14 tumours started in the lower third of the rectum, 12 in the middle and two in the upper third.

### Safety

Dose-limiting toxicities did not occur at each of the three patients at the 500, 650 and 750 mg m^−2^ twice daily dose levels but were observed at the 825 mg m^−2^ twice daily dose. The first three patients experienced one grade IV diarrhoea with the 825 mg m^−2^ twice daily dose. The next three patients at this dose level also reported one grade IV diarrhoea and 1 dose-limiting hand–foot syndrome. So three further patients were treated at the 750 mg m^−2^ twice daily dose level and no DLT occurred. Therefore, the recommended dose level was 750 mg m^−2^ bid capecitabine, concurrently during radiation and weekly irinotecan (40 mg m^−2^, six times). Another 10 patients were included in the phase II part at this dose level (see Table 2).

The most common toxicity was diarrhoea: grade IV in two patients at a dose level of 825 mg m^−2^ and grade III in nine patients (32%). In addition, four patients (14%) had fever (raised temperature of over 38.5°C), which could be easily controlled with usual antibiotics. One patient in the phase I part died of pneumonia, contracted in the intensive care unit, 2 months after treatment was discontinued early as a result of dose-limiting grade IV diarrhoea. During the phase II part of the study, one patient died as a result of a 5-FU-related sudden cardiac death on day 5 of treatment. There were no grade IV haematological toxicities and the only grade III haematological toxicity was a single case of leucopenia (see Table 2). Other toxicity like oral mucositis, hepatic or renal dysfunction was not observed.

After surgery there was only one anastomotic leakage, treated by reoperation, and one bowel atony after extirpation, treated conservatively reported.

### Resection

Of the 28 patients enrolled, 25 underwent surgery, two patients died and one patient did not have surgery because of systemic progress (peritoneal carcinosis; dose level 500 mg m^−2^ twice daily). Of these 25 patients, an R0 resection was possible in 24 cases (96%); one patient underwent a R1 resection during the first dose level of 500 mg m^−2^ (he received additional low-volume radiotherapy in combination with capecitabine and irinotecan and, as of June 2005, he is free of distant metastases and local recurrences). Of the 14 patients with tumours in the lower third (0–5 cm), seven patients had sphincter involvement, so five (36%) underwent sphincter-sparing surgery, the remaining nine patients underwent abdominoperineal resection. Of the patients with tumours in the middle third of the rectum (5.1–10 cm), one required abdominoperineal resection, while the others were able to undergo sphincter-sparing surgery (Table 3).

### Response

Downstaging for the T category was analysed in 25 patients, who underwent surgery (Table 4), and response was analysed in the 26 patients who were alive (including one patient who did not undergo surgery due to systemic progress). Overall, four patients (15%) had pathological complete response (pCR), and further three cases (12%) had only minimal microfocal residual disease (MRD). Pathological partial response (pPR) was achieved in additional 16 patients (62%). In one patient, disease progression was systemic (peritoneal carcinosis) rather than local at the 500 mg m^−2^ twice daily dose level. pCRs were observed at the 750 mg m^−2^ twice daily dose level (three cases) and 825 mg m^−2^ twice daily level (one case); the MRD cases arose at 650, 750 and 825 mg m^−2^ (one case at each dose level; Table 5). It is noteworthy that all the pCR and MRD cases occurred in patients with an initial haemoglobin value above the median value of 13.5 g dl^−1^. The length of the tumour did not affect the pCR and MRD, nor did the uT or uN stage.

### Disease control and survival

All the patients who underwent surgery are alive after a median observation period of 24 months. One R0 resection patient with his tumour in the lower third had a local recurrence after 7 months, one patient had metastases in the liver, which had not been seen during imaging and were diagnosed during surgery. Another patient developed liver metastases after 18 months. One patient developed metastases in the lung after 6 months, another after 15 months. As a result of the short follow-up period the rating of statistical analysis for survival must be with care. The DFS (30 months) for all patients who underwent surgery is 78% (±8%), the local control 96% (±4%), the OS 100%.

## DISCUSSION

Preoperative chemoradiation has the potential to increase resectability and improve local control in patients with LARC. In addition, it can enable patients to undergo more conservative sphincter-sparing surgery and there is a lower rate of acute side effects (Sauer *et al*, 2004). Up to now, it is not known if preoperative chemoradiation had to be intensified with capecitabine, irinotecan or oxaliplatin for better survival data. So the remission rates are used to compare the different studies of preoperative-intensified chemoradiotherapy, and also knowing, that a complete remission to radiation or chemoradiation is associated with favourable survival (Janjan *et al*, 2001).

The rate of complete remission is about 10% by using 5-FU or capecitabine concurrent to radiotherapy (Dunst *et al*, 2002; Sauer *et al*, 2004), by adding irinotecan or oxaliplatin the rate of complete remission in phase II studies can be more than doubled (overview by Klautke *et al*, 2005).

Other studies investigate the combination of preoperative capecitabine and irinotecan concurrent to radiotherapy (Table 6), too. The average dose of irinotecan during the period of radiotherapy was in most studies about 240–250 mg m^−2^. Looking at the capecitabine dose and application time, there were more differences between these studies. So Hofheinz *et al* (2005) applicates 500 mg m^−2^ bid of capecitabine (day 1–38; total dose of capecitabine: 38000 mg m^−2^) and reported a high rate of pCR (4/19; 21%) and MRD (5/19; 26%), and a R0 resection rate of 100%. Gollins (Gollins *et al*, 2005) applicates 825 mg m^−2^ bid from day 1 to 33 continuously (total dose of capecitabine: 54450 mg m^−2^) together with four times irinotecan (60 mg m^−2^). After surgery there was a R0 resection in 94% and a pCR in 20% (4/20). At this dose level there was no DLT. In our study, we saw DLT at a dose of six times irinotecan (40 mg m^−2^) and capecitabine 825 mg m^−2^ bid from day 1–43 (total dose of capecitabine in the dose level with DLT: 70950 mg m^−2^; total dose of capecitabine in the recommended dose level: 64500 mg m^−2^) and we saw the DLT mostly in the fifth week. The rate of pCR (15%) and R0 – resection seems to be comparable with these other studies.

In nearly all these studies diarrhoea was the DLT. The haematological toxicity is not common, in these regimes, and also oral mucositis was not reported. The hand–foot syndrome was also no common toxicity at the different recommended doses of capecitabine. Comparing these results with the results of the combination 5-FU as continuous infusion and irinotecan weekly concurrently to radiotherapy (Mitchell *et al*, 2001; Mehta *et al*, 2003; Klautke *et al*, 2005) there were no real differences in efficacy and toxicity. So the rate of pCR was about 24–37%, and diarrhoea was the most common toxicity with grade III about 30%. Mitchell reported also intravenous catheter infections and thrombi as DLT. And these catheter complications are the big disadvantage of continuous infused 5-FU and the big advantage of capecitabine.

It is a great discussion whether oxaliplatin or irinotecan is the best partner in the preoperative chemoradiotherapy together with capecitabine and radiation and unfortunately there is no effort for a randomized trial to clear this discussion. The rate of complete response does not differ using oxaliplatin or irinotecan, but critics mark the high rate of diarrhoea as disadvantage to irinotecan. Analysing at the study from Duck *et al* (2004) using 825 mg m^−2^ capecitabine bid on each day of radiation, and weekly 50 mg m^−2^ oxaliplatin concurrent to radiation, the most frequent grade III/IV toxicity was diarrhoea in 22%. The pCR was 6% (1/17). Using continuous infused 5-FU over the whole time of radiation together with oxaliplatin concurrent to radiotherapy, diarrhoea also is a DLT (Loi *et al*, 2005), or was reported in 16% by a pCR of 28% (7/25) (Aschele *et al*, 2005).

The preoperative chemoradiotherapy with capecitabine 750 mg m^−2^ bid, concurrently during radiation (days 1–43) and weekly irinotecan 40 mg m^−2^ (six times) seems to be safe and effective. We need a longer follow-up to verify a potential benefit for survival in comparing with historical collectives of patients.

## Figures and Tables

**Table 1:** Baseline patient characteristics

	**Patients (*N*=28)**	
**Variable**	**No.**	**%**
*Gender*		
Male	12	43
Female	16	57
		
*Age (years)*		
Range	48–75	
Median	64	
		
*T stage*		
uT2 (uNo/uN+)	2 (0/2)	7
uT3 (uNo/uN+)	18 (2/16)	64
uT4 (uN0/uN+)	8 (1/16)	29
		
*N stage*		
uN0	3	11
uN+	25	89
		
*Tumour localization (cm from anal verge)*		
0–5	14	50
5.5–10	12	43
>10	2	7
		
*Tumour length (cm)*		
Range	3–15	
Median	7	
		
*Initial Hb value*		
<13.5 g dl^−1^	13	46
⩾13.5 g dl^−1^	15	54

**Table 2:** Inicdence and maximum toxicity grade (CTC) according to capecitabine dose levels

			**Phase I**		**Phase II**
**Capecitabine dose**	**500 mg m^−2^ bid**	**650 mg m^−2^ bid**	**750 mg m^−2^ bid**	**825 mg m^−2^ bid**	**750 mg m^−2^ bid**
Toxicity grade (CTC)	1 2 3 4	1 2 3 4	1 2 3 4	1 2 3 4	1 2 3 4
Number of patients	3	3	3+3	3+3	10
					
*Hematologic*					
Anemia	3 0 0 0	3 0 0 0	5 1 0 0	6 0 0 0	8 2 0 0
Leucopenia	3 0 0 0	3 0 0 0	4 2 0 0	3 2 1 0	4 6 0 0
Thrombopenia	3 0 0 0	3 0 0 0	6 0 0 0	4 2 0 0	9 1 0 0
					
*Laberatory*					
Hyperbilirubinaemia	3 0 0 0	3 0 0 0	6 0 0 0	6 0 0 0	10 0 0 0
ALAT/ASAT	3 0 0 0	3 0 0 0	6 0 0 0	6 0 0 0	10 0 0 0
					
*Gastrointestinal*					
Nausea/vomiting	3 0 0 0	3 0 0 0	4 2 0 0	5 1 0 0	8 2 0 0
Diarrhoea	0 3 0 0	0 3 0 0	0 2 4 0	0 1 3 2	0 8 2 0
					
*Other*					
Hand–foot syndrome	3 0 0 0	3 0 0 0	6 0 0 0	5 0 1 0	10 0 0 0
Infection	3 0 0 0	2 1 0 0	5 1 0 0	5 1 0 0	9 1 0 0

One patient died of pneumonia in the intensive care unit, after a complete resolved diarrhoea grade IV in the phase I, 2 months after treatment had interrupted; one patient died as a result of a 5-FU related sudden cardiac death on day 5 of treatment in the phase II.

**Table 3:** Surgical approach by tumour height

	**Surgical approach (no. patients)**		
**Tumour heigt (cm)**	**Sphincter – saving**	**Abdominop. Resection**	**Total**
0–5	5	9	14
5.5–10	11	1	12
>10	2	0	2

**Table 4:** Pathologic downstaging of the primary tumor

	**Pathological T stage at time of surgery (patients)**					
**Clinical T stage at baseline**	**pT0**	**pT1**	**pT2**	**pT3**	**pT4**	**Total**
cT2	0	1	1	0	0	2
cT3	4	0	6	6	0	16
cT4	2	0	2	3	0	7
Total	6	1	9	9	0	25

**Table 5:** Pathohistological response of 25 patients underwent surgery depending on the dose level of capecitabine

	**Phase I**				**Phase II**
	**Capecitabine dose (bid)**				
**Pathohistological response**	**500 mg m^−2^**	**650 mg m^−2^**	**750 mg m^−2^**	**825 mg m^−2^**	**750 mg m^−2^**
NC	2	0	0	0	0
pPR	0	2	5	3	6
MRD	0	1	0	1	1
pCR	0	0	1	1	2
					
Total	2	3	6	5	9

**Table 6:** Overview of phase I or phase I/II studies with preoperative chemoradiotherapy using capecitabine and irinotecan concurrent to radiation

**Study**	**No of patients**	**Capecitabine (mg m^−2^ bid)**	**Irinotecan (mg m^−2^/week)**	**Radiotherapy (Gy)**	**pCR (%)**	**pCR+MRD**	**R0**	**DLT**
Hofheinz *et al* (2005)	19	500/625	50	45+5,4	21	4+5/19	19/19	Diarrhoea
		Day 1–38	5 ×					
								
Gollins *et al* (2005)	20	650/825	50/60/70	45	20	4+4/20	16/17	Diarrhoea
		Day 1–33	4 ×					Neutrope nic fever
								
Becerra *et al* (2005)	12	850	30/40/50	45+5,4				Study is ongoing
		Day 1–5; 5,5 weeks	5 ×					
								
Kennedy *et al* (2004)	12	500/650/800	40	45+5,4	8	1+2/12	12/12	Study is ongoing
		Day 1–5; 5,5 weeks	4 ×					
								
Present study	28	500/650/750/800	40	50,4+5,4	15	4+3/26	24/25	Diarrhoea
		Day 1–43	6 ×					Hand–feet syndrome

The underlined dose is the recommended dose level.
